# Polyphenols, Antioxidant, Antibacterial, and Biofilm Inhibitory Activities of Peel and Pulp of *Citrus medica* L., *Citrus bergamia*, and *Citrus medica* cv. Salò Cultivated in Southern Italy

**DOI:** 10.3390/molecules24244577

**Published:** 2019-12-13

**Authors:** Florinda Fratianni, Autilia Cozzolino, Vincenzo De Feo, Raffaele Coppola, Maria Neve Ombra, Filomena Nazzaro

**Affiliations:** 1 Istituto di Scienze dell’Alimentazione, Consiglio Nazionale delle Ricerche (ISA-CNR), via Roma 64, 83100 Avellino, Italy; fratianni@isa.cnr.it (F.F.); marianeve.ombra@isa.cnr.it (M.N.O.); 2 Department of Agricultural, Environmental and Food Sciences, DiAAA-University of Molise, Via de Sanctis s.n.c., 83100 Campobasso, Italy; a.cozzolino@studenti.unimol.it (A.C.); coppola@unimol.it (R.C.); 3 Department of Pharmacy, University of Salerno, Via Giovanni Paolo II, 132, 84084 Fisciano (Salerno), Italy

**Keywords:** *Citrus medica*, *Citrus bergamia*, *Citrus medica* cv. Salò, polyphenols, biofilm, antibacterial activity

## Abstract

The aim of this paper was to study the polyphenols of peel and pulp of three *Citrus* taxa—*Citrus medica, Citrus bergamia*, and *Citrus medica* cv. Salò—cultivated in the Cosenza province, Southern Italy, and to evaluate their antioxidant and antibacterial activity, performed against *Escherichia coli*, *Listeria monocytogenes*, *Pseudomonas aeruginosa*, *Staphylococcus aureus*, and *Pectobacterium carotovorum*. Furthermore, we assessed the inhibitory effect of the extracts on bacterial capacity to form biofilm, and on the metabolic activity of the cells present therein. The results indicated that such extracts could find new potential applications in the field of natural antioxidant and anti-bacterial agents in pharmaceutics, agriculture, and food fields.

## 1. Introduction

In recent decades, the international market has been characterized by increased consumer demand for products with improved quality and safety features. Fruit and vegetables contain very precious metabolites with antioxidant activity, which also represent a defense strategy of the plant organism in fighting biotic attacks from pathogenic microorganisms and from chemical-physical stresses due to environmental conditions. Several studies have shown that continuous treatment with conventional antibiotics lead to the development of bacterial resistance [1]. Biofilm formation and bacterial antibiotic resistance constitute problems of particular relevance for human health. In fact, the emergence of antibiotic resistance among pathogenic microorganisms, also through the formation of biofilm, is encouraging research to identify new antimicrobials from various sources, such as medicinal plants that could be capable to act as alternative treatments to commercial antimicrobial drugs, mainly in the case of particularly resistant and aggressive bacteria [2]. The Mediterranean area is rich in vegetal biodiversity and, since ancient times, fruits, herbs, spices, and vegetables have been used in traditional medicine to treat several diseases, including infections caused by bacteria and fungi. Some of the most diffused products, present on both sides of the Mediterranean Sea, are represented by species of genus *Citrus*, belonging to the Rutaceae family [3]. In particular, *Citrus medica* L., the cedar, was introduced to Italy by Hebrews, who promoted its cultivation on the Calabrian and Amalfi coasts and Garda Lake [4,5]. The different cultivars of *C. medica* L. are generally divided into two groups: “acidic” and “sweet” cedars. The first one, like the typical species, have red-purple flowers and buds and acidic pulp; the latter have white flowers and a sweeter pulp. Among the acidic cedars there are *C. medica* cv. *Diamante*, and the so-called “Hand of Buddha” (with ornamental fruits without pulp). The Corsican and the *C. medica* cv. Salò are among the sweet cedars. Finally, other cultivars can be considered as hybrids between lemons and cedars; they produce fruits resembling cedar due to the size and thickness of the peel, quite suitable for candy manufacture, while the appearance is similar to a lemon.

The Calabria region is famous for the production of different *Citrus* species, such as cedar, cedar of Salò, and bergamot. Cedar (*Citrus medica* L.) is also known because it is considered the progenitor of many of the *Citrus* fruits present in the world. The fruit, a large 20–30 cm, is yellowish, oval, or almost round, sometimes with a slight protuberance at the peduncle and a little pointed at the opposite side. Its peel is very rough and exceptionally thick and represents up to 70% of the fruit, so, even after removing the seeds and the film between the segments, only 25%–30% of the cedar is edible. In Calabria, cedar is cultivated and worked mainly in the coastal area of the high Tyrrhenian coast of the province of Cosenza, called “Riviera dei Cedri”. Cedar is used in the food industry for consumption as a table fruit, and for the preparation of soft drinks and candy fruit, but mostly it is consumed in the pharmaceutical industry for the production of the essential oil. Cedar of Salò (*Citrus medica* cv. Salò) is used for the production of essence and in the production of commercial juices. It gives, unlike cedar, a completely clear juice, so that many agronomists choose a total abandonment of the cultivation of cedar in favor of those of cedar of Salò. Bergamot (*Citrus bergamia* Risso) produces a yellow citrus fruit, which—according to some scientists—could be considered as a subspecies of bitter orange or a hybrid derived from bitter orange and lemon. Such products are some of the symbols of excellence of the Calabrian agricultural tradition and represent a great resource for the benefit of the local economy. Bergamot is used almost exclusively for the production of the essential oil, obtained from its peel, and the pulp is considered a merely by-product. On the contrary, the pulp and its juice are a rich source of polyphenols, in particular flavonoids, with attraction for pharmaceutical fields, while searching for new forms of nutraceuticals with beneficial properties for human health [6]. The consumption of polyphenols-rich products, such as *Citrus* consumption, is associated with a reduced cancer incidence [7]. Flavonoids from *Citrus* have also antiviral and anti-inflammatory activities [8,9,10]. They can act beneficially on capillary fragility and inhibit or limit human platelet aggregation [10,11,12,13]. The antimicrobial activity originating from components of these species is well founded. The essential oils of different *Citrus* species act as potent antibacterial agents against several Gram-negative and Gram-positive bacteria [14,15]. Flavonoids present in peel of bergamot exhibit antibacterial activity [16]. Further, bergamot juice also exhibited noticeable activity against *Helicobacter pylori* [17]. However, at least to our knowledge, no papers report the evaluation of the capability of the polyphenolic extracts of these three taxa to inhibit or limit the formation of biofilm by different pathogenic bacteria, nor their effect on bacterial metabolism.

Therefore, the aim of this paper was to study the chemical composition of the extracts obtained from juice and pulp of three *Citrus* taxa cultivated in the above-mentioned “Riviera dei cedri” and to evaluate their antibacterial activity. In particular, we assessed the inhibitory effect of the bacterial capability to form biofilm and to limit the metabolic activity of biofilm cells, in order to exploit new potential applications of such products, with novel challenges in the field of natural anti-bacterial agents.

## 2. Results and Discussion

### 2.1. Chemical Composition of the Extracts

#### 2.1.1. Total Polyphenol Content 

Total polyphenol content (TPC) of the ethanolic extracts of peel and pulp of bergamot, cedar, and cedar of Salò ranged between 148.98 µg/g (pulp of cedar) and 1002.3 µg/g of fresh product (peel of cedar of Salò). By the whole, the cedar of Salò exhibited—both in peel and in pulp—the highest amount of polyphenols, reaching even 1 mg/g of fresh product (Table 1).

This indicates the high healthy valence of this product, whose traditional cultivation has been lost over the centuries and is now, at least in Italy, limited to few areas, such as its original location, Calabria, and to the area surrounding the Lake of Garda, and which could represent a product with wide potentialities for the Italian agri-food economy. Similarly, bergamot possesses noticeable nutritional challenge: peel and pulp, in fact, showed 748 and 208 µg gallic acid equivalent (GAE)/g of TPC, respectively, indicating that the consumption of just one fruit, which weight ranges generally between 80 and 200 g, can provide the body with a considerable amount of polyphenols. Concurrently, cedar, although containing the lowest amount of total polyphenols both in peel and in pulp, represents proportionally the richest source of total polyphenols among the three taxa analyzed (the cedar fruit can be much heavier than that of bergamot, ranging between 500–600 g and even to 1.5–2 kg).

The amount of polyphenols detected in the peel of the three *Citrus* could be considered higher than that found in other *Citrus* species [18]. Polyphenolic content present in cedar was also different compared to that reported by Menichini et al. [19], who detected in the mature fresh fruit of *C. medica* 123.1 mg of total polyphenols in 100 g of the mesocarp. However, this discrepancy may be due, more than to the different cultivation area, to the climatic conditions, which are known to influence the metabolism of these molecules within the product.

#### 2.1.2. Antioxidant Activity

A strong correlation (Correlation-coefficient = 0.96) between the total polyphenol content and the antioxidant activity exhibited by the peel of all three samples analyzed was observed (Table 1). From this point of view, cedar of Salò was once again extremely interesting: in fact, the value of EC_50_ exhibited by its peel extract was just 3965 mg. Cedar, on the other hand, seemed a much weaker antioxidant agent, so much so that it takes almost 20 mg to inhibit by 50% the activity of 1 mL of the 2,2-diphenyl-1-picrylhydrazyl (DPPH) radical. Peel of bergamot showed a good antioxidant activity, although not equal to that exhibited by the peel of cedar of Salò, with an EC_50_ value of 6.47 mg. The values found for bergamot peel, but above all for that of cedar di Salò, can suggest their use as new sources of natural antioxidants, usable as functional ingredients in the food industry. The powder obtained from the entire bergamot fruit has shown, after all, an interesting antioxidant activity in both in vitro and ex vivo studies, which have also shown its ability to limit the endothelial alterations by reducing the resulting stress on the endoplasmic reticulum [20]. Such indications are in line with literature data, which confirm that the essential oil from the bergamot peel has high antioxidant and pro-cholinesterase activities and could be used as a “nutraceutical flavor” for the formulation of food products or nutraceuticals, with particular reference to dietary supplements for elderly populations [21]. While several studies ascertained the biochemical and health characteristics of the bergamot fruit and its different portions, the literature is lacking with regard to cedar of Salò, whose peel has shown remarkable antioxidant activity, as well as the presence of interesting polyphenolic metabolites, with known biological and health activities. Our study confirmed not only the nutraceutical potential of the bergamot pulp but once again underlined the nutraceutical potential of cedar of Salò. The scenario resulting from the analysis of the antioxidant activity of the pulp of the three *Citrus* samples is slightly different. In this case, both the bergamot and the Cedar of Salò pulps exhibited almost the same efficacy in inhibiting 50% of the activity of the DPPH radical. Once again, cedar, also with regard to the pulp, showed a doubly weaker antioxidant activity (Table 1). However, it should be emphasized that the value found for the pulp of the cedar, although lower also than that resulting from other studies [22], is a respectable value, since it refers to the fresh product, and not to a lyophilized or otherwise heat-treated product.

#### 2.1.3. UPLC Profile

Table 2 reported the UPLC polyphenol profile of the extracts.

The three *Citrus* always contained more total phenolic acids than flavonoids. In some cases, for example in the pulp of cedar and cedar of Salò, such an amount was almost five times higher with respect to that of flavonoids. The parts of the three *Citrus* analyzed showed a different amount of phenolic acids. Peel of bergamot exhibited a content of total phenolic acids, which was more than double if compared to what was found in the pulp. Indeed, the peel of Salò cedar contained almost three times more acid phenols than the pulp. Cedar represented an exception, as previously indicated. Its peel had a much lower content of acid phenols (28.67%) than those found in the pulp. Cedar showed a lower content of phenolic acids. Indeed, its profile was characterized by the absence of chlorogenic, caffeic, and p-coumaric acids. On the other hand, ferulic acid was detected only in the pulp. Cedar of Salò showed all the phenolic acids identified. In general, we found a greater quantity of individual polyphenols in the peel with respect to the pulp. In some cases, such as that of rutin, the quantity of the same molecule found in the peel was almost 6 times higher than that found in the pulp (115.47 and 19.39 µg/g of fresh product, respectively). The presence of such an important concentration of rutin in the peel of cedar (where this compound represented 39% of the total polyphenols) is very significant. Rutin (3′,4′,5,7-tetrahydroxy-flavone-3-rutinoside) has been extensively studied due to its anti-inflammatory [23], antibacterial [24], anti-depressant [25], and cancer chemo-preventive [26] activities. Due to its anti-inflammatory properties, rutin has been widely used in the treatment of chronic venous insufficiency; further uses have been suggested for the treatment of glaucoma, hay fever, hemorrhoids, varicose veins, poor circulation, oral herpes, cirrhosis, stress, low serum calcium, and cataracts [27,28]. Rutin is generally poorly absorbed; however, the use of appropriate natural solvents can improve its absorption and solubilization in the organism. This allows a greater recovery of the molecule, so as to open new frontiers in the field of nutraceuticals [29]. In other cases, some flavonoids were present only in the peel (e.g., quercetin in bergamot; catechin in cedar; quercetin and apigenin in cedar of Salò). Only in one case did we find the presence of a metabolite (epicatechin) in the pulp but not in the peel (of the cedar). Therefore, Karoui and Marzouk reported a higher amount of such a metabolite in juice than in the peel of *C. aurantium* L. [30]. Among the three *Citrus* analyzed, cedar of Salò showed the most variegated profile and almost all the known standards available were found both in the pulp and above all in the peel. In cedar of Salò, it is to highlight above all the abundance of the two hydroxycinnamic derivatives, chlorogenic and ferulic acids. The latter was the most abundant metabolite present in the peel (295 µg/g of fresh product), representing 62% of the total phenolic acids. The presence of such a high amount of ferulic acid was in agreement with other studies [30,31]. Two flavonoids, epicatechin (105.1 µg/g of fresh product) and quercetin (which, with 150.89 µg/g of fresh product alone, represented 15% of the total polyphenols present in that portion of the fruit and more than 50% of the total flavonoids identified through UPLC) were present mainly in the peel of cedar of Salò. In this fruit, the relative abundance of the two hydroxycinnamic derivatives, chlorogenic and ferulic acids, as well as of the two well-defined flavonoids, can be highlighted with the degree of maturation of the fruit. The presence of apigenin, but not of naringenin (a common secondary metabolite in *Citrus* fruits), could indicate a high degree of ripeness of the analyzed products. Apigenin, in fact, in the process of biosynthesis of phenylpropanoids, originates from naringenin, which on the one hand leads to the formation of apigenin and on the other gives rise to genistein [32]. Moreover, the high degree of ripeness of the fruits may also explain the significant content of epicatechin, found in the peel and pulp of bergamot and in cedar of Salò. The high ferulic acid content, also found in the other two fruits, and an almost complete absence of caffeic acid (found only in cedar of Salò in very low amounts), confirmed the maturation of the analyzed samples, always keeping in mind the biosynthetic pathway of phenylpronoids and the enzymatic–metabolic steps leading to the synthesis of ferulic acid from caffeic acid [33]. The presence of epicatechin found only in the pulp of the cedar, which is also the only sample presenting rutin, indicated a probable different metabolic pathway in these samples, which led, in the cedar, to the predominance of a flavonoid, rutin, in the peel, and of a hydroxycinnamic acid, ferulic acid, in the pulp.

### 2.2. Biological Activity

#### 2.2.1. Antibacterial Activity

In general, all the six extracts exhibited antibacterial activity against the strains tested. Less effective antibacterial activity was exhibited, just against two strains (*P. carotovorum* and *Ps. aeruginosa*), by the polyphenolic extract obtained from the bergamot pulp (Table 3). 

These results are in agreement with previous studies reporting the effectiveness of action of polyphenols from *Citrus* versus both Gram-negative and Gram-positive strains [3,12,14]. Concerning cedar of Salò, we usually found a slightly greater efficacy in inhibiting microbial growth exhibited by the extract of the peel compared to that shown by the extract of the pulp. Peel of cedar was slightly more effective than pulp against *L. monocytogenes* and *Ps. aeruginosa*. Of particular importance the apparently good antibacterial behavior of the pulp extracts of all three samples versus EHEC *E. coli* and, that of the pulp of bergamot, against *Staph. aureus*. The presence of such a variegated pattern of polyphenols within the extracts could be considered important, also due to their influence on colonic microbiota, with positive effects on human health [34,35,36,37]. The data obtained could stimulate new studies about the effects of bergamot whole-fruit on selected beneficial or pathogenic gut bacteria that, to the best of our knowledge, appear little studied. This corroborates, once again, with the potentialities of the polyphenols of the pulp of *Citrus* in restricting the growth of pathogenic bacteria. Moreover, their possible use in food processes, as natural preserving agents, appears of importance. At same time, the effectiveness of some of the extracts to inhibit the growth of the phytopathogenic strain, *P. carotovorum,* could open new opportunities in the treatment of plants with natural antibiotics.

#### 2.2.2. Biofilm Formation and Metabolic Activity of Biofilm Cells

**Figure 1:**
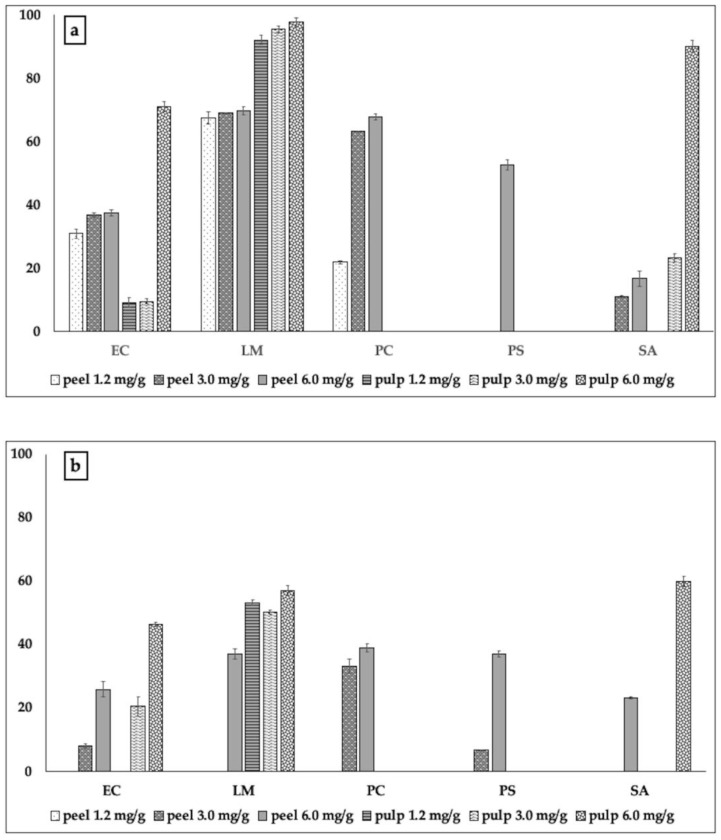
Inhibitory action of the extracts from pulp and peel of bergamot on the bacterial capability to form biofilm (**a**) and on the metabolic activity of bacterial cells present within the biofilm (**b**). The test was performed using three sub-minimal inhibitory concentrations of the samples, previously calculated through the resazurin test for each tester pathogenic strain used in the experiments. The data are reported as percentage with respect to the control. Legend: EC: *E. coli*; LM: *L. monocytogenes*; PC: *P. carotovorum*; PS: *Ps. aeruginosa*; SA: *Staph. aureus.*

Polyphenols present in the peel of bergamot showed, in general, the ability to inhibit the formation of biofilms by the pathogenic strains used (Figure 1a). 

**Figure 2:**
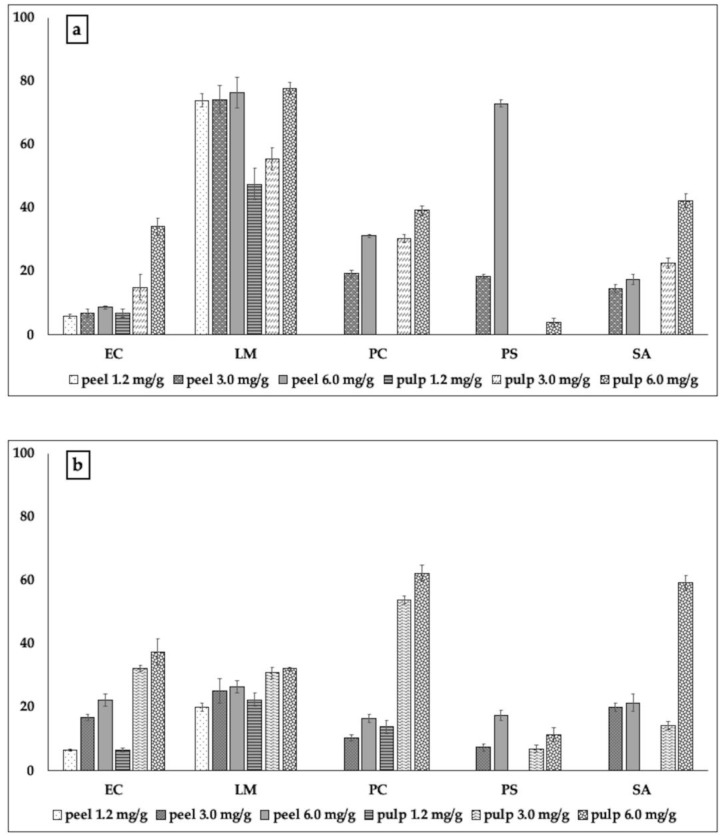
Inhibitory action of the extracts from peel and pulp of cedar, on the bacterial capability to form biofilm (**a**) and on the metabolic activity of bacterial cells present within the biofilm (**b**). The test was performed using three sub-minimal inhibitory concentrations of the samples, previously calculated through the resazurin test for each tester pathogenic strain used in the experiments. The data are reported as percentage with respect to the control. Legend: EC: *E. coli*; LM: *L. monocytogenes*; PC: *P. carotovorum*; PS: *Ps. aeruginosa*; SA: *Staph. aureus.*

They were active against all pathogens tested, although with different effectiveness. Polyphenols of the pulp of bergamot did not exhibit a wider spectrum of action; they were almost incapable of constraining the biofilm formation by *P. carotovorum* and *Ps. aeruginosa*. However, in the other cases, they were much more effective with respect to those of peel, inhibiting the formation of biofilm almost completely (97.61%). In the case of *L. monocytogenes*, they inhibited 92.02% of the biofilm, just with 1.2 mg; in addition, 6 mg of such extract inhibited up to 90% of the biofilm formation by *Staph. aureus.* Such capability also corroborated with the data concerning the inhibitory action on viability of the cells present inside the biofilm, which was always more effective with respect to that exhibited by polyphenols of peel against *E.coli*, *L. monocytogenes*, and *Staph. aureus* (Figure 1b). At the same time, we noted almost total inactivity of the pulp extract against *P. carotovorum* and *Ps. aeruginosa*. The polyphenolic extract of bergamot peel, although less effective, proved to be active in inhibiting the formation of biofilm by *P. carotovorum* (up to 67.72% inhibition) and *Ps. aeruginosa* (52.5% inhibition). The test with MTT showed that the extracts of bergamot peel were not only able to inhibit the formation of biofilm, but also to act on the metabolism of the cells therein, Figure 1b). The behavior exhibited by the polyphenols of cedar was in some cases similar, although with less efficacy (Figure 2a,b). 

**Figure 3:**
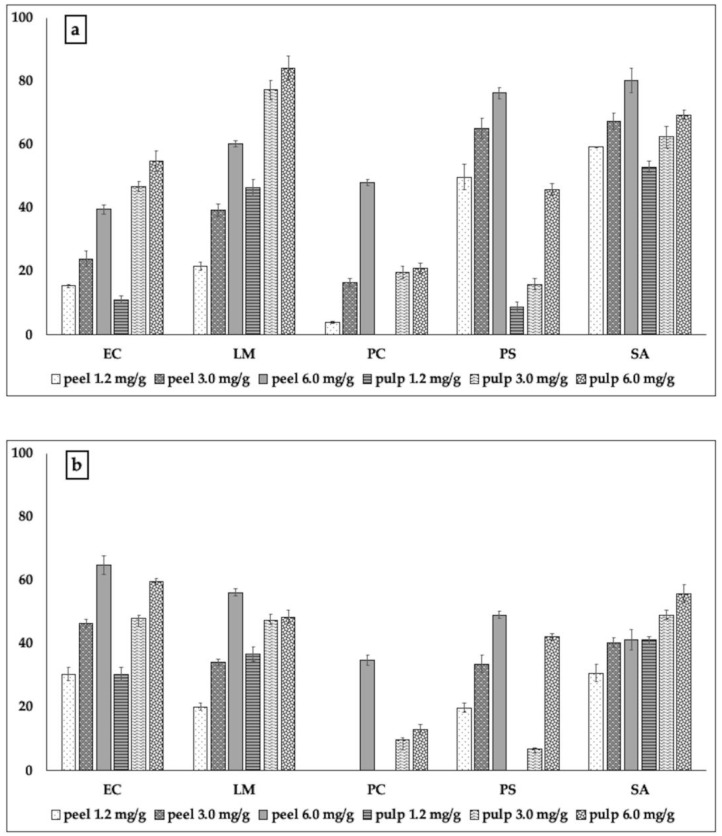
Inhibitory action of the extracts from peel and pulp of cedar of Salò, on the bacterial capability to form biofilm (**a**) and on the metabolic activity of bacterial cells present within the biofilm (**b**). The test was performed using three sub-minimal inhibitory concentrations of the samples, previously calculated through the resazurin test for each tester pathogenic strain used in the experiments. The data are reported as percentage with respect to the control. Legend: EC: *E. coli*; LM: *L. monocytogenes*; PC: *P. carotovorum*; PS: *Ps. aeruginosa*; SA: *Staph. aureus.*

Only with regard to *P. aeruginosa*, the inhibitory activity exhibited by cedar peel was much stronger than that exerted by the pulp of cedar at the same quantities of TP used in the experiments (72.9% and 3.6%, respectively, Figure 2a). Pulp of cedar seemed more effective than the peel in inhibiting the formation of biofilm by *E. coli* and *Staph. aureus*; this was also reflected in the worse metabolic activity exhibited by the cells of these two strains within the respective biofilms (Figure 2b). Cedar of Salò, in contrast, was overall active and effective in inhibiting biofilm formation (Figure 3). 

Also in this case, as already observed for cedar and bergamot, the pulp of cedar of Salò, at the highest of the sub-MIC concentrations used, was more effective than the peel against *L. monocytogenes* and *E.coli*, and was able to inhibit the metabolic activity of the cells up to 59.7% (against *E. coli,* Figure 3a). The action of most of the extracts in blocking both biofilm formation and the metabolic activity of biofilm bacterial cells of the phytopathogen *P. carotovorum* (Figure 3b) confirmed that such extracts could also find interesting applications in agriculture.

The data acquired showed that the polyphenolic extracts obtained from the two portions (peel and pulp) of the three *Citrus* taxa were generally able not only to limit the formation of biofilm by pathogenic bacteria, but also to alter the metabolic activity of the cells present within the biofilm, thus counteracting the formation of biofilm on several fronts, including that of acting on the metabolism of biofilm bacterial cells. This confirms the great and interesting applicative versatility of *Citrus*, whose species can provide extracts or essential oils capable of preventing or at least reducing, the pathogenicity of different bacteria, acting through different mechanisms of action [38]. The presence of flavonoids in the extracts appears to modulate the mechanisms of cellular communication. From the analysis of the polyphenols, however, we could hypothesize that the inhibitory activity against the biofilm and the metabolism of bacterial biofilm cells was due not only to the presence of flavonoids, but also to the presence of phenolic acids, in particular chlorogenic and ferulic acids, whose capacity in this sense has been demonstrated by Virkam et al. [39]. Moreover, phenolic acids, such as chlorogenic [40] and ferulic acids [41], are able to prevent and limit the formation of biofilms. In the case of cedar, there is a further consideration linked to the presence of rutin, which was reported for its inhibitory power on the biofilm formation of *P. aeruginosa* [42]. Here, the presence of rutin meant that especially peel of cedar was able to inhibit the formation of biofilm by *Ps. aeruginosa* with a percentage equal to 76.3%, and to act on the vitality and metabolism of the cells present in the biofilm, which turned out to be viable and active but much less than half (inhibition = 42.2%) compared to untreated bacterial cells.

## 3. Materials and Methods 

### 3.1. Materials

Caffeic, ferulic, *p*-coumaric, gallic, and chlorogenic acids, rutin, catechin, quercetin, 2,2-diphenyl-1-picrylhydrazyl (DPPH), 3-(4,5-dimethylthiazol-2-yl)-2,5-diphenyl tetrazolium bromide (MTT), dimethylsulphoxide (DMSO), sterile Luria-Bertani broth, tetracycline, ciprofloxacin, HPLC-grade ethanol, 4-methyl-2-pentanol, ethanol, and acetonitrile were purchased from Sigma-Aldrich (Milano, Italy). Apigenin was purchased from Extrasynthese (Genay, France). Ultrapure water was produced by a Milli-Q system (Millipore, Milan, Italy). 

#### Plant Material

Fruits of *Citrus medica* L. (cedar), *C. bergamia* Risso (bergamot), and *Citrus medica* cv. Salò (cedar of Salò) were collected in 2015 in S. Maria del Cedro (Cosenza, 39°45′N 15°50′E), Southern Italy. Professor Vincenzo De Feo identified the plants and provided to store voucher specimens in the Herbarium of the Medical Botany Chair at the University of Salerno. 

### 3.2. Biochemical Characterization

Biochemical characterization involved the analysis of the total polyphenol content, the antioxidant activity, and the qualitative–quantitative profile of polyphenols by ultra-pressure liquid chromatography.

#### 3.2.1. Polyphenol Extraction

Peel was removed from fruits, carefully avoiding the recovery of the albedo, pounded, and left in contact with ethanol 100% (1:2 *v/v*) for 24 h in the dark at 4 °C. Pulp was homogenized with a domestic homogenizer and, after centrifugation for 5 min at 3000 rpm (Beckman Italia Cassina de’ Pecchi, Milan, Italy), the juice was recovered and centrifuged again for 20 min at 6000 rpm. Then, it was mixed with two volumes of 100% ethanol, and left at 4 °C for 24 h.

#### 3.2.2. Total Polyphenol Content

The dosage of total polyphenols present in the ethanolic extracts was carried out following the protocol of Singleton and Rossi [43], using the Folin-Ciocalteau reagent. The absorbance was measured at room temperature at λ = 760 nm, with a Vary Cary UV/Vis spectrophotometer (Varian Cary Spectrophotometer model 50 MPR, Cernusco sul Naviglio, MI, Italy). The quantification of the total polyphenols was obtained through a calibration curve generated with gallic acid. Results were expressed in terms of concentration (µg) of GAE/gr of fresh sample.

#### 3.2.3. Antioxidant Activity

The antioxidant activity of the extracts was determined by evaluating their capability to inhibit the activity of the 2,2-diphenyl-1-picrylhydrazyl radical (DPPH test) [44]. The analysis was carried out on microtiter plates, by adding 7.5 μL of the samples to 303 μL of a solution of DPPH in methanol (153 mM) and measuring the absorbance λ = 517 nm (Varian Cary Spectrophotometer model 50 MPR, Cernusco sul Naviglio, MI, Italy). The antioxidant activity was expressed in terms of EC_50_, representing the effective concentration capable of inhibiting the DPPH radical activity of 50% after a 60-min incubation. All experiments were performed in triplicate. The correlation with respect to total polyphenols was calculated through the program Excel (Microsoft, Redmond, WA, USA).

#### 3.2.4. Polyphenol profile

The analysis of the polyphenolic profile was carried out following the method of Fratianni et al. [45]. An ultra-pressure liquid chromatograph with diode array detector (UPLC-DAD) (Waters Italia, Sesto San Giovanni (MI), Italy) was used for the analysis; data acquisition was carried out with the Empower Waters software. The analyses were performed at 30 °C using a BEH C_18_ column, 1.7 μm, 2.1 mm × 100 mm. The mobile phase consisted of 7.5 mM acetic acid (eluent A) and acetonitrile (eluent B). The analyses were conducted at a back-pressure value between 6000 and 8000 psi. The DAD detector was programmed to a scanning range between 210 and 400 nm, with a resolution of 1.2 nm. The injection volume was 5 μL. Quantification of the polyphenols was determined through calibration curves obtained from the polyphenol standards mentioned above.

### 3.3. Antibacterial Activity

#### 3.3.1. Microorganisms and Culture Conditions

*Staphylococcus aureus* ATCC 25923, *Listeria monocytogenes* ATCC 7644, EHEC, *Escherichia coli* DSM 8579, *Pseudomonas aeruginosa* DSM 50071, and the phytopathogen *Pectobacterium carovotorum* DSM 102074 were used as test bacterial strains. We previously stored the strains at −30 °C in sterile Luria Bertani (LB) broth (Sigma, Milano, Italy) supplemented with 20% sterile glycerol (Sigma, Milano, Italy). Bacteria were thawed and added (inoculum 2%) to LB broth. *S. aureus, L. monocytogenes*, *E.coli*, and *P. aeruginosa* were grown for 18 h at 37 °C and 80 rpm (Corning LSE, Pisa, Italy). *P. carotovorum* was grown at 30 °C and 80 rpm. Then, fresh cultures were used as inoculum (2% final concentration) and grown in the conditions above described.

#### 3.3.2. Minimal Inhibitory Concentration (MIC)

Values of the Minimal Inhibitory Concentration (MIC) were calculated using the resazurin microtiter-plate assay [46,47]. Two-fold serial dilutions were performed to have 50 µL of the test material in serially descending concentrations in each well. A 35 μL amount of 3.3 × strength iso-sensitized broth and 5 μL of resazurin, used as indicator solution, were added to reach a final volume/well of 240 μL with different volumes of sterile Luria-Bertani broth previously set. Lastly, 10 μL of bacterial suspension was added to each well to reach a concentration of about 0.5 McFarland (1.5 × 10^7^ cells/mL) (Densitometer cell density turbidity 0.3–15.0 McFarland, CAMLAB, Cambridge, UK). Sterile DMSO and ciprofloxacin (prepared dissolving 1 mg/mL in DMSO) were used as negative and positive control, respectively. Microtiter-plates were prepared in triplicate and incubated at 37 °C for 24 h. The lowest concentration at which a color change occurred (from dark purple to colorless) indicated the MIC value.

#### 3.3.3. Biofilm Inhibitory Activity

The biofilms of *S. aureus*, *L. monocytogenes E. coli*, *P. aeruginosa*, and *P. carovotorum* were formed in flat-bottomed 96-well microtiter plates following the method of O’Toole and Kolter [48] using sub-MIC values. In each well, the overnight bacterial cultures were adjusted to 0.5 McFarland (1.5 × 10^7^ cells/mL) (Densitometer cell density turbidity 0.3–15.0 McFarland, CAMLAB, Cambridge, UK) using Luria-Bertani broth. Then, 10 µL of the diluted cultures were distributed in each well, and different volumes of the extracts and Luria-Bertani broth were added, to reach a final volume of 200 µL/well. Microplates were completely covered with parafilm, to avoid the evaporation of samples with relative loss of volume and incubated for 48 h. Planktonic cells were removed and the attached cells were gently washed twice with sterile physiological saline solution. Then, 200 µL of methanol/well, which was left for 20 min to fix the sessile cells, was added. The methanol was discarded, and the plates were left under laminar flow cap until complete dryness of samples (at least 30 min). The staining of the adhered cells was obtained by adding 200 µL of 2% *w/v* crystal violet solution to each well that was left for 20 min. Wells were gently washed and left to dry. A volume of 200 µL of glacial acetic acid 20% *w/v* was added to allow the release of the bound dye. The absorbance was measured at OD = 540 nm (Varian Cary). Evaluation of the biofilm inhibition was calculated (percentage) with respect to that of control (cells grown without the presence of the extracts). Triplicate tests were done, and the average results were taken for reproducibility.

#### 3.3.4. Metabolic Activity of Biofilm Cells

The effect of the extracts from bergamot, cedar, and cedar of Salò on the metabolic activity of cells present in the biofilm was evaluated through the MTT colorimetric test, modifying the method of Kairo et al. [49] as follows. After 48 h of bacterial growth in the 96-well microtiter plates, planktonic cells were removed and 150 µL of PBS and 30 µL of 0.3% MTT were added. Then, microtiter plates were kept at 37 °C. After 3 h, the MTT solution was removed and 200 µL of DMSO/well were added, to allow the dissolution of the formazan crystals formed in the wells. After 3 h incubation at 37 °C, resulting purple formazan derivatives were dissolved in DMSO and measured at OD = 570 nm (Varian Cary). Triplicate tests were done, and the average results were taken for reproducibility. 

## Figures and Tables

**Table 1:** Total polyphenols and antioxidant activity exhibited by the ethanolic extracts obtained from peel and pulp of bergamot, cedar, and cedar of Salò. Total polyphenols are reported as µg gallic acid equivalent (GAE)/gr of fresh product, antioxidant activity was expressed in terms of EC_50_, representing the effective concentration (mg) capable of inhibiting the 2,2-diphenyl-1-picrylhydrazyl (DPPH) radical activity of 50% after a 60-min incubation period.

	Total Polyphenols (µg GAE/g of Fresh Product ± SD)			Antioxidant Activity (DPPH Test, EC_50_)		
	Bergamot	Cedar	Cedar of Salò	Bergamot	Cedar	Cedar of Salò
**peel**	748.08 ± 43.67	291.97 ± 20.81	1002.3 ± 54.41	6.47 ± 0.48	19.40 ± 1.15	3.96 ± 0.09
**pulp**	208.02 ± 14.55	148.98 ± 14.51	242.73 ± 15.17	10.99 ± 0.58	24.22 ± 1.62	10.46 ± 1.03

**Table 2:** Polyphenol composition of the ethanolic extracts from peel and pulp of bergamot, cedar, and cedar of Salò. The data are reported as average of µg/g of fresh product ± standard deviation. Legend = nd: not detected.

	Bergamot		Cedar		Cedar of Salò	
	Peel	Pulp	Peel	Pulp	Peel	Pulp
**Acidic phenols**						
Gallic acid	24.68 ± 1.03	54.186 ± 2.34	39.02 ± 2.10	22.16 ± 1.55	33.49 ± 1.37	16.84 ± 1.67
Chlorogenic acid	71.98 ± 1.67	28.91 ± 0.57	nd	nd	116.39 ± 6.32	45.3 ± 2.14
Caffeic acid	nd	nd	nd	nd	7.11 ± 0.57	6.97 ± 0.57
Coumaric acid	nd	nd	nd	nd	21.05 ± 0.57	11.61 ± 0.57
Ferulic acid	181.58 ± 6.32	48.30 ± 1.67	nd	113.91 ± 1.67	295.97 ± 10.2	106.36 ± 6.32
**Flavonoids**						
Rutin	nd	nd	115.47 ± 5.42	19.39 ± 1.67	nd	nd
Epicatechin	73.40 ± 1.67	26.87 ± 1.67	nd	9.85 ± 0.57	105.1 ± 1.67	44.4 ± 1.67
Quercetin	97.32 ± 10.2	nd	nd	nd	150.89 ± 6.32	nd
Apigenin	nd	nd	nd	nd	24.26 ± 1.67	nd
Catechin	20.56 ± 2.14	7.03 ± 0.57	4.34 ± 0.57	nd	68.78 ± 1.37	11.84 ± 1.37
**Total phenolic acids**	278.24	131.40	39.02	136.07	474.01	187.08
**Total flavonoids**	191.28	33.9	119.81	29.24	349.03	56.24
**Total phenolic compounds**	469.52	165.3	158.83	169.31	823.04	243.32

**Table 3:** Minimal inhibitory concentration of the polyphenol extracts from the pulp and peel of bergamot, cedar, and cedar of Salò evaluated through the resazurin test, as reported in the Materials and Methods.

MIC (mg/mL)	Bergamot		Cedar		Cedar of Salò	
	Peel	Pulp	Peel	Pulp	Peel	Pulp
***E. coli***	9.00	8.00	10.00	9.00	9.00	8.00
***L. monocytogenes***	8.00	7.00	7.00	8.00	8.00	7.00
***P. carotovorum***	8.00	>10.00	9.00	9.00	9.00	10.00
***Ps. aeruginosa***	8.00	>10.00	7.00	10.00	8.00	9.00
***Staph. aureus***	10.00	7.00	10.00	9.00	7.00	8.00
